# Generational differences in associations between health conditions in young women and BMI categories

**DOI:** 10.1002/oby.24304

**Published:** 2025-05-13

**Authors:** Annette J. Dobson, Chen Liang, Gita D. Mishra

**Affiliations:** ^1^ Australian Women and Girls' Health Research Centre, School of Public Health University of Queensland Brisbane Queensland Australia

## Abstract

**Objective:**

The objective of this study was to investigate whether the associations between BMI categories and the age‐specific prevalence of health conditions common in young women differed across generations.

**Methods:**

Data were from the Australian Longitudinal Study on Women's Health for participants born between 1973 and 1978 or 1989 and 1995 and recruited in 1996 and 2013, respectively. Women were included if they reported their weight and height at waves conducted when they were aged 18 to 23, 22 to 27, and 25 to 30 years. Outcomes were self‐rated health, the prevalence of common conditions, menstrual symptoms, and pregnancy complications. Odds ratios (ORs) were estimated using logistic regression models with generalized estimating equations to account for repeated measures.

**Results:**

For fair or poor self‐rated health, the ORs were higher for women in the underweight range (OR 1.51, 95% CI: 1.30–1.74) or the overweight range (OR 1.47, 95% CI: 1.34–1.60), were highest for women with obesity (OR 3.04, 95% CI: 2.76–3.35) compared with women with normal weight, and were higher for the more recent cohort (OR 1.50, 95% CI: 1.38–1.63). This same pattern was apparent for all outcomes.

**Conclusions:**

The health impacts of increasing BMI are not lessened in more recent generations. This evidence can be used to promote the benefits of normal BMI for young women.

**Study Importance**

**What is already known?**

- BMI is increasing across generations in many countries.
- Life expectancy and health‐adjusted life expectancy are increasing in most countries.

**What does this study add?**

- The odds of poorer health and menstrual symptoms were higher for young women in the underweight or overweight range compared with normal weight and were highest for women with obesity.
- This U‐shaped pattern was apparent in two different cohorts defined by year of birth, but the odds were higher in the more recent cohort.

**How might these results change the direction of research or the focus of clinical practice?**

- The findings do not support the hypothesis that the health impacts of increasing BMI are less in more recent generations.
- This evidence can be used to promote the health benefits of normal BMI for young women.

## INTRODUCTION

The prevalence of overweight and obesity is increasing worldwide, especially among women [1, 2]. In adults, having overweight (including obesity) is a major risk factor for cardiovascular diseases, diabetes, cancers, neurological disorders, chronic respiratory diseases, and digestive disorders [3]. Nevertheless, over the past few decades, prior to 2019 and the COVID‐19 pandemic, life expectancy at birth and health‐adjusted life expectancy increased in most countries [4, 5].

Against this background of increasing body mass index (BMI) and increasing healthy life expectancy, there are influences acting to normalize overweight and obesity and overlook the health consequences. One comes from the scientific interest in the concept of “fat and fit,” i.e., the concept that there are people with high levels of both BMI and metabolic fitness [6, 7, 8]. Most of the evidence for or against this concept relates to cardiometabolic risk among adolescents and young people [9], whereas the noncommunicable diseases commonly associated with having BMI in the overweight and obesity ranges are diagnosed in middle‐aged and older adults [10]. This leaves the effects of increasing levels of overweight and obesity on the health of younger adults, including women in their reproductive years, understudied. The normalization of overweight and obesity has been amplified in the popular media and the “body positivity” movement, which aims to reduce the stigma associated with obesity [11, 12]. Nevertheless, there are calls for the longitudinal investigation of the health outcomes of body positivity [13].

In order to understand the effects of increasing BMI on health, several factors need to be considered. First, BMI tends to increase with age until the age of ~60 years [14, 15]. Second, BMI has increased across generations in many populations [15, 16, 17, 18]. Third, medical diagnosis and treatments change over time, leading to improvements in population health and decreases in many noncommunicable diseases. Therefore, in order to examine the associations between trends in BMI and health conditions, it is necessary to take into account the effects of age, differences between generations (or birth cohorts), and temporal changes that affect everyone. One approach is to compare the associations between BMI categories and health conditions over time between different generations in the same population over the same age range.

The Australian Longitudinal Study on Women's Health (ALSWH) provides an opportunity to undertake such an analysis. In this paper we focus on conditions that affect young women and girls for whom relatively little is known about associations between health and overweight and obesity [19, 20]. Specifically, we investigate the null hypothesis that the associations between BMI categories and common conditions, menstrual symptoms, and pregnancy complications remain constant across generations.

## METHODS

The ALSWH is a prospective, nationwide, population‐based study that started in 1996 by recruiting women in three cohorts, i.e., those who were born between 1973 and 1978, 1946 and 1951, and 1921 and 1926 [21]. Participants were randomly selected from the Medicare database (the universal health care system for Australian citizens and permanent residents). A fourth cohort of women born between 1989 and 1995 was recruited in 2013 via the internet and social networking sites [22]. The women were broadly representative of the general population of Australian women of the same ages at the time of recruitment, although, owing to attrition and population changes, they are now more likely to be Australian‐born and have higher educational levels than the general population. Further details are available on the ALSWH website (www.alswh.org.au). The study was approved by the Human Research Ethics Committees of the Universities of Queensland and Newcastle in Australia. All women provided informed consent.

The current analyses included data from participants born between 1989 and 1995 or 1973 and 1978 and recruited 17 years apart. Data were collected using self‐report surveys in repeated waves conducted every 1 to 3 years. The waves selected were those completed by women who were of the same ages, i.e., 18 to 23, 22 to 27, and 25 to 30 years (referred to as waves 1–3). BMI, defined as weight in kilograms divided by height in meters squared, was categorized as follows: underweight (BMI < 18.5), normal weight (18.5 ≤ BMI < 25), overweight (25 ≤ BMI < 30), and obesity (BMI ≥ 30) [23]. Sociodemographic variables were the highest level of educational attainment, area of residence, marital status, and ability to manage on available income, which were all measured at every wave, and language spoken at home, which was collected at baseline.

The choice of health conditions for this analysis was driven by several factors. Questions regarding the condition had to be included in at least five of the six waves (e.g., polycystic ovary syndrome could not be included because it was not mentioned in early waves for the 1973–1978 cohort). The condition needed to be sufficiently common, even in the youngest age groups, to enable statistically valid comparisons to be made between BMI categories and cohorts; this ruled out most diagnosed conditions that are often associated with higher BMI values at older ages (e.g., hypertension, type 2 diabetes), which were uncommon in women aged less than 30 years. Conditions were included if the prevalence was at least 5% in most BMI categories at all waves. Conditions were not included if they were likely to predate young adult BMI and unlikely to lead to weight gain, e.g., asthma was most likely to be diagnosed first in childhood. Using these criteria, the conditions considered were self‐rated health from the 36‐Item Short‐Form Survey (SF‐36) scale [24]; the following symptoms in the last 12 months: depression, back pain, leaking urine, irregular periods, heavy periods, severe period pain, and premenstrual tension; having ever had a diagnosis of endometriosis; and cumulative incidence of miscarriage.

The self‐rated health question is “In general would you say your health is …?” with response options “excellent,” “very good,” “good,” “fair,” or “poor,” which were categorized as “excellent/very good/good” and “fair/poor.” For symptoms, the women were asked “Have you had any of the following …?” with response options of “never,” “rarely,” “sometimes,” and “often,” which were dichotomized as “often” compared to “never/rarely/sometimes” (except for leaking urine, in which case “sometimes” and “often” were combined). Comparable questions on depression were not asked in wave 1 for the 1973–1978 cohort; therefore, these data were missing. At each wave, participants were asked “Have you been diagnosed with or treated for…?” or “Have you been told by a doctor you have …?” for a range of conditions and various time frames; the responses were used to identify those with a history of endometriosis. The women were also asked how many times they had been pregnant and how many times they had a miscarriage [25].

Potential confounding variables for the associations health conditions include smoking (classified as “never smoked,” “ex‐smoker,” or “current smoker”), physical activity (calculated as metabolic equivalent of a task multiplied by the number of minutes doing the task [MET‐min] per week and categorized as “nil/sedentary,” <33.3 MET‐min; “low,” 33.3 to <500 MET‐min; “moderate,” 500 to <1000 MET‐min; or “high,” ≥1000 MET‐min), and ever having been pregnant. Comparable measures of physical activity were not available from wave 1 of the 1973–1978 cohort; therefore, these data were missing. The sociodemographic covariates varied with age and differed substantially among the cohorts; therefore, to reduce confounding and help with interpretation of the results, wave (a proxy for age at completion of each wave) and cohort (i.e., born between 1973 and 1978 or 1989 and 1995) were included in the model, and the cohort‐related demographics were not included.

The distributions of participant characteristics and health conditions tabulated by cohort, wave, and BMI categories are presented as percentages. Logistic regression was used to estimate odds ratios (ORs) for various health conditions across BMI categories, with terms for wave and cohort in the basic models. Population average estimates were calculated using generalized estimating equations to account for the repeated measures of the same individuals across waves (with exchangeable correlations and robust standard errors) [26]. For most of the health conditions, there was evidence of interaction between BMI and cohort; therefore, additional models were fitted with terms for all combinations of BMI categories and cohort. Owing to the missing data on depression and physical activity at wave 1 for the 1973–1978 cohort, multiple imputation using chained equations was conducted for all of the outcomes, with all other variables used in the models included in the imputation (i.e., cohort, wave, BMI categories, physical activity, smoking, and ever having been pregnant). Stata version 18 (StataCorp LLC) was used for all analyses.

## RESULTS

There were *N* = 6060 women in the 1973–1978 cohort and *N* = 6231 women in the 1989–1995 cohort who provided height and weight data in all three relevant waves, i.e., the analysis sample (Table 1). The main reason for the difference between the initial number of participants and the analysis sample was attrition, especially after wave 1 (Table S1). The proportions of participants with missing data needed for BMI categorization were 9% to 13% across waves for the 1973–1978 cohort and 4% to 7% across waves for the 1989–1995 cohort. The distributions of BMI categories at each wave were similar for all women who participated in that wave and those in the analysis sample (Table S2).

**TABLE 1:** ALSWH waves used for this analysis: dates, ages and numbers of participants, and sociodemographic characteristics for women who provided height and weight data at all waves.

Cohort	Born 1973–1978			Born 1989–1995		
Wave, year of data collection	1, 1996	2, 2000	3, 2003	1, 2013	2, 2017	3, 2019
Age range at this wave, y	18–23	22–27	25–30	18–23	22–27	24‐30^a^
Number of participants in each wave	14,247	9688	9081	17,010	8495	8346
Number of participants in all waves^b^	6060			6231		
Age at wave, mean (SD), y	20.78 (1.45)	24.57 (1.47)	27.55 (1.46)	20.70 (1.68)	24.70 (1.75)	26.76 (1.78)
Highest level of education, %						
School	68.8	31.2	25.7	49.1	14.2	9.6
Trade	17.1	23.5	24.6	22.3	23.4	21.1
University	14.1	45.4	49.8	28.7	62.4	69.3
Area of residence, %						
Major city	53.1	53.4	58.9	75.2	74.9	74.7
Inner regional area	29.9	29.8	25.2	17.3	16.7	17.0
Other regional area	14.1	14.0	13.0	6.4	6.8	6.7
Remote	2.9	2.8	2.9	1.2	1.6	1.6
Language spoken at home, %^c^						
English	92.6	x	x	97.6	x	x
Other	7.4	x	x	2.4	x	x
Marital status, %						
Married/living together	18.7	41.5	59.2	22.1	42.5	52.4
Single/widowed/divorced/separated	81.3	58.5	40.8	77.9	57.5	47.6
Ability to manage on income, %^c^						
Impossible/difficult	15.8	x	10.2	20.8	14.4	12.8
Sometimes difficult	30.9	x	28.6	35.5	30.6	26.6
Not too bad	38.6	x	39.4	30.8	37.1	38.3
Easy	14.7	x	21.8	12.9	17.8	22.3

Abbreviation: ALSWH, Australian Longitudinal Study on Women's Health.

^a^ Owing to the COVID‐19 pandemic, responses to wave 3 (2003) for the 1989–1995 cohort were returned over a longer period; therefore, the age range is wider.

^b^ Women who provided data on height and weight in all three waves.

^c^ Question not asked in this wave, indicated by “x.”

For both cohorts the highest level of educational achievement increased across the waves as the women moved from high school and gained trade or university qualifications. Overall, education levels were lower in the 1973–1978 cohort than the 1989–1995 cohort at the same age. Owing to recruitment differences, women in the 1973–1978 cohort were more likely to live in regional areas than major cities, but, for both cohorts, there was little change in area of residence across waves. In both cohorts, most women spoke English at home (and few were born in non‐English‐speaking countries, data not shown). With increasing age, more women were married or living with partners and fewer were single in both cohorts. Consistent with the increasing levels of education and changing marital status across waves, the women's ability to manage on their available income also improved, and there was little difference between the cohorts. (There were similar differences for those women who participated in all waves included in this analysis and those who participated in any of the relevant waves; data not shown.).

BMI increased in both cohorts as they aged, but women in the 1973–1978 cohort were less likely to be in the overweight or obesity category (Table 2). The 1973–1978 cohort participants were more likely to be current smokers and had lower levels of physical activity. They rated their general health higher and were less likely to report experiencing symptoms than the 1989–1995 cohort (except for premenstrual tension). As expected, more women reported having ever been pregnant across successive waves, and, consistent with national trends of increasing age at first birth, the 1973–1978 cohort participants were more likely than the 1989–1995 cohort to have ever been pregnant at comparable ages.

**TABLE 2:** Health‐related data for women who reported their height and weight in all waves.

Cohort	Born 1973–1978			Born 1989–1995		
Wave	1	2	3	1	2	3
BMI category, %						
Underweight (BMI < 18.5)	9.3	6.4	4.6	7.2	3.7	2.8
Normal weight (18.5 ≤ BMI < 25)	67.8	63.3	59.0	63.2	54.1	49.5
Overweight (25 ≤ BMI < 30)	16.3	19.8	22.2	18.6	23.5	25.4
Obesity (BMI ≥ 30)	6.7	10.5	14.2	11.0	18.8	22.3
Smoking, %						
Never smoked	58.4	59.8	60.1	69.8	77.2	76.6
Ex‐smoker	14.3	13.4	16.9	17.5	9.8	11.2
Current smoker	27.4	26.8	23.0	12.8	13.0	12.2
Physical activity, %						
Nil/sedentary (<33.3 MET‐min)	x	8.6	8.0	5.3	5.6	7.3
Low (33.3 ≤ MET‐min < 500)	x	30.8	30.2	23.2	23.6	25.9
Moderate (500 ≤ MET‐min < 1000)	x	24.3	23.9	22.3	22.1	22.5
High (MET‐min ≥ 1000)	x	36.4	37.9	49.2	48.7	44.3
Self‐rated health, %						
Excellent	13.6	13.9	14.3	8.4	10.1	10.4
Very good	41.1	40.2	42.8	40.6	39.5	41.5
Good	34.4	34.6	34.2	37.1	36.5	34.2
Fair/poor	10.9	11.4	8.7	13.9	13.9	13.9
Symptoms in last 12 mo, %						
Depression (often)	x	6.9	6.0	16.1	13.1	12.4
Back pain (often)	10.5	12.9	11.7	18.6	16.7	17.0
Leaking urine (sometimes/often)	4.6	6.1	6.4	10.3	11.9	13.2
Irregular periods (often)	11.5	8.5	8.7	18.0	19.3	19.4
Heavy periods (often)	8.8	6.0	6.5	14.1	12.0	12.4
Severe period pain (often)	16.3	10.5	9.4	19.6	14.9	14.0
Premenstrual tension (often)	16.8	14.0	11.8	16.4	14.9	15.8
Diagnosis of endometriosis	1.6	4.4	6.1	3.4	6.9	9.1
Ever been pregnant	12.1	24.0	35.9	7.0	18.1	26.4
Miscarriages, Br % of pregnancies	9.1	15.1	14.2	21.1	9.3	10.5

Abbreviation: MET‐min, metabolic equivalent of task multiplied by minutes.

Averaged over the three waves, the prevalence of health conditions showed a U‐shaped pattern with BMI categories, i.e., higher for women with BMI in the underweight and overweight ranges than in the group with normal weight and highest among women with obesity (Table 3). The shape of the pattern was similar in both cohorts, but the prevalence was generally higher in the 1989–1995 cohort.

**TABLE 3:** Health variables by BMI categories and cohorts (averaged across all waves).

BMI category	Born 1973–1978				Born 1989–1995			
	Underweight	Normal weight	Overweight	Obesity	Underweight	Normal weight	Overweight	Obesity
Self‐rated health (fair/poor), %	12.3	8.2	10.6	21.7	15.8	8.7	14.7	29.2
Depression (often in last 12 mo), %	7.4	5.8	6.8	8.7	15.8	11.2	13.6	21.9
Back pain (often in last 12 mo), %	12.4	11.0	12.6	13.7	17.3	14.7	18.5	25.0
Leaking urine (sometimes/often in last 12 mo), %	5.1	4.8	6.5	9.6	10.1	9.6	11.7	19.4
Irregular periods (often in last 12 mo), %	11.7	8.9	8.3	14.3	22.8	16.6	17.5	26.7
Heavy periods (often in last 12 mo), %	6.4	6.1	8.7	10.7	10.0	10.3	13.0	21.5
Severe period pain (often in last 12 mo), %	17.1	11.2	12.5	13.4	16.0	13.9	16.5	22.9
Premenstrual tension (often in last 12 mo), %	20.3	13.6	14.3	13.8	15.2	14.4	16.6	18.8
Diagnosis of endometriosis (ever), %	4.1	3.7	4.4	4.9	5.1	5.4	7.3	9.3
Miscarriages, as % of pregnancies	14.8	13.1	12.6	17.2	15.2	10.9	11.2	13.2

**FIGURE 1:**
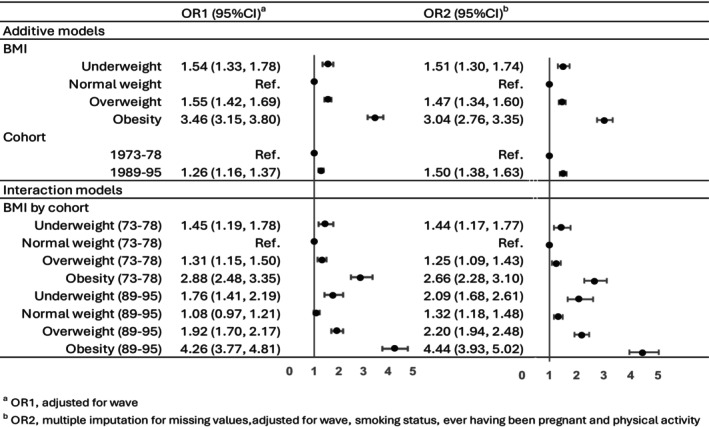
Odds ratio (OR) estimates (95% CI) for the associations between fair/poor self‐rated health and categories of BMI, taking into account the effects of cohort, wave, smoking status, ever having been pregnant, and physical activity.

This pattern persisted when the ORs were calculated using various models. The effect is illustrated for fair or poor self‐rated health in Figure 1 and Table S3. From the additive model with multiple imputation for missing values and adjustment for all of the covariates, the ORs for poorer self‐rated health were higher for the 1989–1995 cohort compared with the 1973–1978 cohort (OR 1.50, 95% confidence interval [CI]: 1.38–1.63). Compared with women in the normal‐weight category, the ORs were higher for women in the underweight category (OR 1.51, 95% CI: 1.30–1.74) or overweight category (OR 1.47, 95% CI: 1.34–1.60) and highest for women with obesity (OR 3.04, 95% CI: 2.76–3.35). The odds of poorer self‐rated health declined across waves, i.e., self‐rated health improved as the women became older for wave 2 and wave 3 (OR 0.92, 95% CI: 0.86–0.99 and OR 0.77, 95% CI: 0.69–0.81, respectively) compared with wave 1. The odds of poorer self‐rated health were higher for ex‐smokers and current smokers (OR 1.20, 95% CI: 1.09–1.33 and OR 1.83, 95% CI: 1.67–2.00, respectively) than for women who never smoked. The odds were  hardly affected by having ever been pregnant (OR 1.08, 95% CI: 0.99–1.19). However, the odds of fair or poor self‐rated health decreased as the level of physical activity increased: compared with women in the moderate physical activity category, the ORs for the sedentary and low physical activity categories were 1.95 (95% CI: 1.71–2.22) and 1.30 (95% CI: 1.19–1.42), respectively, and for women in the high physical activity category, the OR was 0.68 (95% CI: 0.62–0.75).

Similar tables for all other health outcomes are provided in the online Supporting Information, and the results are summarized in Table 4. With some minor differences, the same pattern was apparent for depression, back pain, leaking urine, and all the menstrual symptoms: women in the 1989–1995 cohort and those with obesity had the highest odds of each condition. However, age (or, equivalently, reproductive life stage) was the most important factor for endometriosis and miscarriage. Being a current or ex‐smoker was associated with higher risk for all conditions. Ever having been pregnant was also associated with increased risk, although the effect was small for self‐rated health and depression. Lower levels of physical activity were associated with poorer self‐rated health, depression, and back pain, but not with other conditions or menstrual symptoms.

**TABLE 4:** Summary of modeling the patterns of associations among health outcomes, BMI categories, and other variables.

	BMI category × cohort	Wave	Smoker	Ever pregnant	Physical activity
Self‐rated health (fair/poor)	U‐shaped, highest for BMI ≥ 30, much higher for 1989–1995 cohort	↓	↑	‐	↓
Depression (often in last 12 mo)	U‐shaped, highest for BMI ≥ 30, much higher for 1989–1995 cohort	↓	↑	‐	↓
Back pain (often in last 12 mo)	U‐shaped, highest for BMI ≥ 30, higher for 1989–1995 cohort	↓	↑	↑	↓
Leaking urine (sometimes/often in last 12 mo)	U‐shaped, highest for BMI ≥ 30, much higher for 1989–1995 cohort	‐	↑	↑	
Irregular periods (often in last 12 mo)	U‐shaped, highest for BMI ≥ 30, much higher for 1989–1995 cohort	↓	↑	↑	
Heavy periods (often in last 12 mo)	U‐shaped, highest for BMI ≥ 30, much higher for 1989–1995 cohort	↓	↑	↑	
Severe period pain (often in last 12 mo)	U‐shaped, highest for BMI ≥ 30, higher for 1989–1995 cohort	↓	↑	↑	
Premenstrual tension (often in last 12 mo)	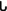 ‐shaped for 1973–78 cohort, U‐shaped for 1989–1995 cohort	↓	↑	↑	
Diagnosis of endometriosis (ever)	N/A for 1973–1978 cohort, J‐shaped for 1989–1995 cohort	↑	↑	↑	
Miscarriages as % of pregnancies	N/A for 1973–1978 cohort, ↓ for 1989–1995 cohort	↑	↑	N/A	

*Note*: BMI category × cohort: U‐shaped indicates odds ratios (ORs) for the condition were higher for women with BMI in the underweight, overweight, and obesity categories compared with the group with normal weight; 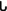
‐shaped indicated ORs were highest for women with BMI in the underweight category; and N/A (not applicable) indicates no significant differences among BMI categories. Wave: ↓ indicates ORs decreased over time in the same cohort; ↑ indicates ORs increased over time; and N/A indicates no differences over time. Smoker: ↑ indicates ORs were higher for ex‐smokers and even higher for current smokers compared with never smokers. Ever pregnant: ↑ indicates ORs were higher for women who had ever been pregnant compared with those who had not; and N/A indicates no differences between women who had been pregnant and those who had not; this factor was N/A for the risk of miscarriage, as this was only estimated as a proportion of pregnancies. Physical activity: ↓ indicates ORs decreased as the level of physical activity increased; and N/A indicates no differences over time.

## DISCUSSION

For most of the conditions considered, risk of poorer health was higher for women who had BMI in the underweight or overweight range compared with those with BMI in the normal‐weight range and was highest for those with obesity. Contrary to the null hypothesis, the pattern did not remain constant (or even decrease) across generations, but rather odds were higher for the 1989–1995 cohort than among women born between 1973 and 1978. For all conditions except for endometriosis and miscarriage, the effect of cohort was greater than the effect of wave (Tables S3–S12). This shows that generational differences were more important than period effects. Prevalence of both endometriosis and miscarriage increases with age (and, thus, wave). For endometriosis, this relates to long times to diagnosis [27], and for miscarriages, risk increases with number of pregnancies.

At least for young women, despite demographic and environmental changes and shifts in the BMI distribution over time, the health consequences of BMI outside the normal‐weight range were the same as or worse for the more recent generation than predecessors. Any decreases seen in cardiometabolic and other chronic conditions in middle and older ages may be attributable to improvements in the social determinants of health, better medical diagnosis and treatments, and reductions in tobacco smoking. Nevertheless, there are concerns that the gains from improvements in medical care will be reduced by the epidemic of overweight and obesity [28].

There are several possible reasons for the poorer health reported by the 1989–1995 cohort across all BMI categories. First, higher levels of education, greater health literacy, and use of technology may have made them more aware of medical conditions [29]. Second, they may have experienced improvements in medical diagnosis or changes in diagnostic criteria. Third, the 1989–1995 cohort was much more likely to live in major urban areas and thereby have better access to medical services. Finally, the greater levels of anxiety and stress experienced by the younger generation could also underlie the propensity to report other conditions [30].

Strengths of this paper include the use of longitudinal cohort data from community samples of women at the same ages but separated by a 17‐year period. The main limitations are that all of the data were all self‐reported, and the samples were recruited by different methods, which led to some of the demographic differences. The validity of self‐reported BMI was assessed using anthropometric measurements from a substudy of 492 women in the 1973–1978 cohort [31]. On average, the self‐reported heights were 0.22 cm higher and the self‐reported weights were 2.78 kg lower than clinically measured values. This resulted in misclassification to lower BMI categories for 16% of women and misclassification to higher BMI categories for 4% of women when self‐reported data were used (unpublished data). The effects of this reporting bias would be to underestimate the strength of the associations between BMI categories and the outcomes.

A particular feature of this paper is that participants were all young women before the ages at which the chronic conditions such as diabetes and hypertension usually associated with overweight and obesity are commonly diagnosed. The conditions considered herein are those typically experienced by women in their 20s and 30s. These were strongly associated with categories of higher BMI values, especially obesity. However, women in the underweight category were also more likely than women of normal weight to experience poorer self‐rated health, depression, back pain, irregular periods, and other menstrual symptoms, especially premenstrual tension. The associations of menstrual problems with low BMI values [32] and high BMI values [33] have been reported previously, and both are also associated with depression [34]. This paper additionally shows that these associations persisted across cohorts.

The public health challenges of overweight and obesity in populations have been well documented [19, 35]. Social and commercial factors contribute to the increasingly obesogenic environment [36, 37, 38]. Additionally, it has been recognized that the stigma of obesity may hamper prevention [11, 12]. For girls and women of reproductive age in particular, a multitude of factors influencing body weight have been documented in high‐income [39] as well as low‐ and middle‐income countries [40]. Against this background, our findings provide strong and highly relevant evidence to support the promotion to young women of health benefits of BMI in the normal‐weight range.

## FUNDING INFORMATION

Australian Government Department of Health and Aged Care funds the Australian Longitudinal Study on Women's Health (ALSWH). Gita D. Mishra is supported by the Australian National Health and Medical Research Council Leadership Fellowship (APP2009577).

## CONFLICT OF INTEREST STATEMENT

The authors declared no conflicts of interest.

## Supporting information


**Data S1.** Supporting Information.

## Data Availability

The datasets analyzed during the current study are available on reasonable request as described here: Australian Longitudinal Study on Women's Health (ALSWH), alswh.org.au/for-data-users/applying-for-data/
