# Supplementary material for: Generational differences in associations between health conditions in young women and BMI categories
Source: Obesity (Silver Spring). 2025 May 13;33(7):1386–94. doi: 10.1002/oby.24304 (PMC12210108; doi:10.1002/oby.24304)
Supplement: Supplementary file 1 — Data S1. Supporting Information. [file OBY-33-1386-s001.docx]

Supplementary materials

Table S1. Number (%) of participants and number with missing BMI data at each wave.

| 1973-78 cohort |  |  |  |
| --- | --- | --- | --- |
|  | Wave 1 | Wave 2 | Wave 3 |
| Number of participants (% of wave 1 participants) | 14247 (100%) | 9688 (68.0%) | 9081 (63.7%) |
| Number with missing BMI (% of participants at this wave) | 1837 (12.9%) | 845 (8.7%) | 977 (10.8%) |
|  |  |  |  |
| 1989-95 cohort |  |  |  |
|  | Wave 1 | Wave 2 | Wave 3 |
| Number of participants (% of wave 1 participants) | 17010 (100%) | 8495 (49.9%) | 8346 (49.1) |
| Number with missing BMI (% of participants at this wave) | 600 (3.5%) | 313 (3.7%) | 586 (7.0%) |

Table S2. Comparison of BMI categories among all participants at each survey and those in the analysis sample

| 1973-78 cohort | | | | | | |
| --- | --- | --- | --- | --- | --- | --- |
|  | Wave 1 | | Wave 2 | | Wave 3 | |
|  | All participants | Analysis sample | All participants | Analysis sample | All participants | Analysis sample |
| N | 14247 | 6060 | 9688 | 6060 | 9081 | 6060 |
| Underweight (%) | 8.6 | 9.3 | 5.9 | 6.4 | 4.0 | 4.6 |
| Normal (%) | 59.4 | 67.8 | 57.3 | 63.3 | 52.1 | 59.0 |
| Overweight (%) | 13.5 | 16.3 | 18.1 | 19.8 | 19.7 | 22.2 |
| Obese (%) | 5.6 | 6.7 | 10.0 | 10.5 | 13.4 | 14.2 |
| Missing (%) | 12.9 | - | 8.7 | - | 10.8 | - |
|  |  |  |  |  |  |  |
| 1989-95 cohort | | | | | | |
|  | Wave 1 | | Wave 2 | | Wave 3 | |
|  | All participants | Analysis sample | All participants | Analysis sample | All participants | Analysis sample |
| N | 17010 | 6231 | 8495 | 6231 | 8346 | 6231 |
| Underweight (%) | 7.6 | 7.2 | 3.7 | 3.7 | 2.6 | 2.8 |
| Normal (%) | 57.4 | 63.2 | 51.2 | 54.1 | 45.8 | 49.5 |
| Overweight (%) | 18.6 | 18.6 | 22.4 | 23.5 | 23.5 | 25.4 |
| Obese (%) | 12.9 | 11.0 | 19.0 | 18.8 | 21.1 | 22.3 |
| Missing (%) | 3.5 | - | 3.7 | - | 7.0 | - |

Table S3. Odds ratios for fair/ poor (compared to excellent/very good/good) Self-Rated Health

|  | Model 1 | Model 2 | Model 3 | Model 4 |
| --- | --- | --- | --- | --- |
| BMI |  |  |  |  |
| Underweight | 1.54 (1.33, 1.78) |  | 1.51 (1.30, 1.75) |  |
| Normal weight | 1 (reference) |  | 1 (reference) |  |
| Overweight | 1.55 (1.42, 1.69) |  | 1.47 (1.34,1.60) |  |
| Obesity | 3.46 (3.15, 3.80) |  | 3.04 (2.76, 3.35) |  |
| Cohort |  |  |  |  |
| 1973-78 | 1 (reference) |  | 1 (reference) |  |
| 1989-95 | 1.26 (1.16, 1.37) |  | 1.50 (1.38, 1.63) |  |
| BMI by cohort |  |  |  |  |
| Underweight (73-78) |  | 1.45 (1.19, 1.78) |  | 1.44 (1.17, 1.77) |
| Normal weight (73-78) |  | 1 (reference) |  | 1 (reference) |
| Overweight (73-78) |  | 1.31 (1.15,1.50) |  | 1.25 (1.09, 1.43) |
| Obesity (73-78) |  | 2.88 (2.48, 3.35) |  | 2.66 (2.28, 3.10) |
| Underweight (89-95) |  | 1.76 (1.41, 2.19) |  | 2.09 (1.68, 2.61) |
| Normal weight (89-95) |  | 1.08 (0.97, 1.21) |  | 1.32 (1.18, 1.48) |
| Overweight (89-95) |  | 1.92 (1.70, 2.17) |  | 2.20 (1.94, 2.48) |
| Obesity (89-95) |  | 4.26 (3.77, 4.81) |  | 4.44 (3.93, 5.02) |
| Wave |  |  |  |  |
| 1 | 1 (reference) | 1 (reference) | 1 (reference) | 1 (reference) |
| 2 | 0.93 (0.87, 1.00) | 0.93 (0.87, 0.99) | 0.92 (0.86, 0.99) | 0.92 (0.86, 0.99) |
| 3 | 0.77 (0.71, 0.82) | 0.77 (0.71, 0.82) | 0.75 (0.69, 0.81) | 0.75 (0.69, 0.81) |
| Smoker |  |  |  |  |
| Never smoked |  |  | 1 (reference) | 1 (reference) |
| Ex-smoker |  |  | 1.20 (1.09, 1.33) | 1.20 (1.09, 1.33) |
| Current smoker |  |  | 1.83 (1.67, 2.00) | 1.83 (1.67, 2.00) |
| Ever pregnant |  |  |  |  |
| No |  |  | 1 (reference) | 1 (reference) |
| Yes |  |  | 1.08 (0.99, 1.19) | 1.09 (0.99, 1.19) |
| Physical activity |  |  |  |  |
| Nil/sedentary |  |  | 1.95 (1.71, 2.22) | 1.94 (1.71, 2.22) |
| Low |  |  | 1.30 (1.19, 1.42) | 1.30 (1.19, 1.42) |
| Moderate |  |  | 1 (reference) | 1 (reference) |
| High |  |  | 0.68 (0.62, 0.75) | 0.68 (0.62, 0.75) |

Model 1: Additive effects for BMI category and cohort, with adjustment for wave.

Model 2: Interaction effects for BMI category and cohort, with adjustment for wave.

Model 3: Additive effects for BMI category and cohort, with multiple imputation for missing values and adjustment for wave, smoking status, ever having been pregnant and level of physical activity.

Model 4: Interaction effects for BMI category and cohort, with multiple imputation for missing values and adjustment for wave, smoking status, ever having been pregnant and level of physical activity.

Table S4. Odds ratios for depression often (compared to never, rarely or sometimes)

|  | Model 1 | Model 4 |
| --- | --- | --- |
| BMI |  |  |
| Underweight | 1.29(1.08, 1.53) |  |
| Normal weight | 1 (reference) |  |
| Overweight | 1.21 (1.10, 1.36) |  |
| Obesity | 3.46 (3.15, 3.80) |  |
| Cohort |  |  |
| 1973-78 | 1 (reference) |  |
| 1989-95 | 1.99 (1.79, 2.20) |  |
| BMI by cohort |  |  |
| Underweight (73-78) |  | 1.09 (0.81, 1.47) |
| Normal weight (73-78) |  | 1 (reference) |
| Overweight (73-78) |  | 1.14 (0.95, 1.37) |
| Obesity (73-78) |  | 1.41 (1.13, 1.75) |
| Underweight (89-95) |  | 2.95 (2.35, 3.69) |
| Normal weight (89-95) |  | 2.30 (2.02, 2.62) |
| Overweight (89-95) |  | 2.73 (2.37, 3.14) |
| Obesity (89-95) |  | 4.29 (3.71, 4.96) |
| Wave |  |  |
| 1 | 1 (reference) | 1 (reference) |
| 2 | 0.75 (0.69, 0.81) | 0.78 (0.71, 0.85) |
| 3 | 0.66 (0.60, 0.72) | 0.68 (0.62, 0.75) |
| Smoker |  |  |
| Never smoked |  | 1 (reference) |
| Ex-smoker |  | 1.64 (1.47, 1.83) |
| Current smoker |  | 2.41 (2.18, 2.66) |
| Ever pregnant |  |  |
| No |  | 1 (reference) |
| Yes |  | 1.04 (0.94, 1.15) |
| Physical activity |  |  |
| Nil/sedentary |  | 1.56 (1.34, 1.82) |
| Low |  | 1.26 (1.13, 1.40) |
| Moderate |  | 1 (reference) |
| High |  | 0.95 (0.86, 1.05) |

Model 1: Additive effects for BMI category and cohort, with adjustment for wave.

Model 4: Interaction effects for BMI category and cohort, with multiple imputation for missing values and adjustment for wave, smoking status, ever having been pregnant and level of physical activity.

Table S5. Odds ratios for back pain often (compared to never, rarely or sometimes)

|  | Model 1 | Model 4 |
| --- | --- | --- |
| BMI |  |  |
| Underweight | 1.13 (1.00, 1.29) |  |
| Normal weight | 1 (reference) |  |
| Overweight | 1.24 (1.15, 1.33) |  |
| Obesity | 1.62 (1.48, 1.78) |  |
| Cohort |  |  |
| 1973-78 | 1 (reference) |  |
| 1989-95 | 1.54 (1.43, 1.66) |  |
| BMI by cohort |  |  |
| Underweight (73-78) |  | 1.10 (0.91, 1.31) |
| Normal weight (73-78) |  | 1 (reference) |
| Overweight (73-78) |  | 1.13 (1.00, 1.28) |
| Obesity (73-78) |  | 1.23 (1.05, 1.44) |
| Underweight (89-95) |  | 1.86 (1.53, 2.25) |
| Normal weight (89-95) |  | 1.63 (1.48, 1.80) |
| Overweight (89-95) |  | 2.00 (1.79, 2.24) |
| Obesity (89-95) |  | 2.64 (2.34, 2.97) |
| Wave |  |  |
| 1 | 1 (reference) | 1 (reference) |
| 2 | 0.98 (0.92, 1.04) | 0.97 (0.91, 1.03) |
| 3 | 0.92 (0.87, 0.98) | 0.89 (0.83, 0.95) |
| Smoker |  |  |
| Never smoked |  | 1 (reference) |
| Ex-smoker |  | 1.43 (1.31, 1.56) |
| Current smoker |  | 1.61 (1.48, 1.75) |
| Ever pregnant |  |  |
| No |  | 1 (reference) |
| Yes |  | 1.30 (1.20, 1.41) |
| Physical activity |  |  |
| Nil/sedentary |  | 1.31 (1.15, 1.48) |
| Low |  | 1.15 (1.05, 1.25) |
| Moderate |  | 1 (reference) |
| High |  | 0.94 (0.87, 1.02) |

Model 1: Additive effects for BMI category and cohort, with adjustment for wave.

Model 4: Interaction effects for BMI category and cohort, with multiple imputation for missing values and adjustment for wave, smoking status, ever having been pregnant and level of physical activity.

Table S6. Odds ratios for leaking urine sometimes or often (compared to never, rarely or sometimes)

|  | Model 1 | Model 4 |
| --- | --- | --- |
| BMI |  |  |
| Underweight | 1.04 (0.86, 1.27) |  |
| Normal weight | 1 (reference) |  |
| Overweight | 1.30 (1.18, 1.43) |  |
| Obesity | 2.07 (1.86, 2.31) |  |
| Cohort |  |  |
| 1973-78 | 1 (reference) |  |
| 1989-95 | 2.08 (1.88, 2.29) |  |
| BMI by cohort |  |  |
| Underweight (73-78) |  | 1.05 (0.78, 1.41) |
| Normal weight (73-78) |  | 1 (reference) |
| Overweight (73-78) |  | 1.33 (1.13, 1.56) |
| Obesity (73-78) |  | 1.89 (1.55, 2.30) |
| Underweight (89-95) |  | 2.40 (1.82, 3.17) |
| Normal weight (89-95) |  | 2.41 (2.11, 2.74) |
| Overweight (89-95) |  | 2.84 (2.46, 3.28) |
| Obesity (89-95) |  | 4.49 (3.87, 5.01) |
| Wave |  |  |
| 1 | 1 (reference) | 1 (reference) |
| 2 | 1.16 (1.07, 1.25) | 1.07 (0.99, 1.16) |
| 3 | 1.23 (1.13, 1.33) | 1.07 (0.98, 1.16) |
| Smoker |  |  |
| Never smoked |  | 1 (reference) |
| Ex-smoker |  | 1.24 (1.10, 1.39) |
| Current smoker |  | 1.28 (1.14, 1.42) |
| Ever pregnant |  |  |
| No |  | 1 (reference) |
| Yes |  | 1.95 (1.77, 2.15) |
| Physical activity |  |  |
| Nil/sedentary |  | 1.19 (1.02, 1.39) |
| Low |  | 1.11 (1.00, 1.23) |
| Moderate |  | 1 (reference) |
| High |  | 1.05 (0.95, 1.15) |

Model 1: Additive effects for BMI category and cohort, with adjustment for wave.

Model 4: Interaction effects for BMI category and cohort, with multiple imputation for missing values and adjustment for wave, smoking status, ever having been pregnant and level of physical activity.

Table S7. Odds ratios for irregular periods often (compared to never, rarely or sometimes)

|  | Model 1 | Model 4 |
| --- | --- | --- |
| BMI |  |  |
| Underweight | 1.34 (1.17, 1.53) |  |
| Normal weight | 1 (reference) |  |
| Overweight | 1.03 (0.95, 1.11) |  |
| Obesity | 1.74 (1.59, 1.91) |  |
| Cohort |  |  |
| 1973-78 | 1 (reference) |  |
| 1989-95 | 2.13 (1.97, 2.29) |  |
| BMI by cohort |  |  |
| Underweight (73-78) |  | 1.28 (1.05, 1.56) |
| Normal weight (73-78) |  | 1 (reference) |
| Overweight (73-78) |  | 0.91 (0.80, 1.05) |
| Obesity (73-78) |  | 1.59 (1.34, 1.89) |
| Underweight (89-95) |  | 2.94 (2.43, 3.55) |
| Normal weight (89-95) |  | 2.15 (1.95, 2.36) |
| Overweight (89-95) |  | 2.28 (2.04, 2.56) |
| Obesity (89-95) |  | 3.73 (3.31, 4.20) |
| Wave |  |  |
| 1 | 1 (reference) | 1 (reference) |
| 2 | 0.91 (0.85, 0.97) | 0.90 (0.85, 0.96) |
| 3 | 0.90 (0.84, 0.96) | 0.89 (0.83, 0.95) |
| Smoker |  |  |
| Never smoked |  | 1 (reference) |
| Ex-smoker |  | 1.15 (1.05, 1.27) |
| Current smoker |  | 1.39 (1.27, 1.51) |
| Ever pregnant |  |  |
| No |  | 1 (reference) |
| Yes |  | 1.13 (1.04, 1.23) |
| Physical activity |  |  |
| Nil/sedentary |  | 1.07 (0.93, 1.23) |
| Low |  | 1.05 (0.96, 1.15) |
| Moderate |  | 1 (reference) |
| High |  | 1.06 (0.98, 1.15) |

Model 1: Additive effects for BMI category and cohort, with adjustment for wave.

Model 4: Interaction effects for BMI category and cohort, with multiple imputation for missing values and adjustment for wave, smoking status, ever having been pregnant and level of physical activity.

Table S8. Odds ratios for heavy periods often (compared to never, rarely or sometimes)

|  | Model 1 | Model 4 |
| --- | --- | --- |
| BMI |  |  |
| Underweight | 0.94 (0.79, 1.13) |  |
| Normal weight | 1 (reference) |  |
| Overweight | 1.35 (1.23, 1.48) |  |
| Obesity | 2.08 (1.87, 2.31) |  |
| Cohort |  |  |
| 1973-78 | 1 (reference) |  |
| 1989-95 | 1.80 (1.64, 1.97) |  |
| BMI by cohort |  |  |
| Underweight (73-78) |  | 1.04 (0.82, 1.33) |
| Normal weight (73-78) |  | 1 (reference) |
| Overweight (73-78) |  | 1.37 (1.18, 1.59) |
| Obesity (73-78) |  | 1.65 (1.35, 2.01) |
| Underweight (89-95) |  | 1.60 (1.23, 2.09) |
| Normal weight (89-95) |  | 1.89 (1.68, 2.13) |
| Overweight (89-95) |  | 2.44 (2.13, 2.78) |
| Obesity (89-95) |  | 4.00 (3.49, 4.60) |
| Wave |  |  |
| 1 | 1 (reference) | 1 (reference) |
| 2 | 0.71 (0.66, 0.76) | 0.70 (0.66, 0.76) |
| 3 | 0.72 (0.67, 0.78) | 0.71 (0.66, 0.76) |
| Smoker |  |  |
| Never smoked |  | 1 (reference) |
| Ex-smoker |  | 1.19 (1.07, 1.32) |
| Current smoker |  | 1.38 (1.25, 1.53) |
| Ever pregnant |  |  |
| No |  | 1 (reference) |
| Yes |  | 1.16 (1.06, 1.28) |
| Physical activity |  |  |
| Nil/sedentary |  | 1.12 (0.96, 1.31) |
| Low |  | 1.03 (0.93, 1.14) |
| Moderate |  | 1 (reference) |
| High |  | 0.96 (0.88, 1.06) |

Model 1: Additive effects for BMI category and cohort, with adjustment for wave.

Model 4: Interaction effects for BMI category and cohort, with multiple imputation for missing values and adjustment for wave, smoking status, ever having been pregnant and level of physical activity.

Table S9. Odds ratios for severe period pain often (compared to never, rarely or sometimes)

|  | Model 1 | Model 4 |
| --- | --- | --- |
| BMI |  |  |
| Underweight | 1.21 (1.05, 1.38) |  |
| Normal weight | 1 (reference) |  |
| Overweight | 1.21 (1.12, 1.31) |  |
| Obesity | 1.59 (1.44, 1.75) |  |
| Cohort |  |  |
| 1973-78 | 1 (reference) |  |
| 1989-95 | 1.35 (1.25, 1.45) |  |
| BMI by cohort |  |  |
| Underweight (73-78) |  | 1.42 (1.19, 1.70) |
| Normal weight (73-78) |  | 1 (reference) |
| Overweight (73-78) |  | 1.12 (0.99, 1.27) |
| Obesity (73-78) |  | 1.20 (1.01, 1.41) |
| Underweight (89-95) |  | 2.09 (1.68, 2.61) |
| Normal weight (89-95) |  | 1.34 (1.22, 1.48) |
| Overweight (89-95) |  | 1.69 (1.51, 1.89) |
| Obesity (89-95) |  | 2.39 (2.11, 2.70) |
| Wave |  |  |
| 1 | 1 (reference) | 1 (reference) |
| 2 | 0.64 (0.61, 0.68) | 0.66 (0.62, 0.70) |
| 3 | 0.57 (0.54, 0.61) | 0.60 (0.56, 0.63) |
| Smoker |  |  |
| Never smoked |  | 1 (reference) |
| Ex-smoker |  | 1.13 (1.03, 1.24) |
| Current smoker |  | 1.47 (1.35, 1.60) |
| Ever pregnant |  |  |
| No |  | 1 (reference) |
| Yes |  | 0.89 (0.81, 0.97) |
| Physical activity |  |  |
| Nil/sedentary |  | 1.05 (0.92, 1.21) |
| Low |  | 0.99 (0.90, 1.08) |
| Moderate |  | 1 (reference) |
| High |  | 0.97 (0.90, 1.05) |

Model 1: Additive effects for BMI category and cohort, with adjustment for wave.

Model 4: Interaction effects for BMI category and cohort, with multiple imputation for missing values and adjustment for wave, smoking status, ever having been pregnant and level of physical activity.

Table S10. Odds ratios for premenstrual tension often (compared to never, rarely or sometimes)

|  | Model 1 | Model 4 |
| --- | --- | --- |
| BMI |  |  |
| Underweight | 1.32 (1.16, 1.48) |  |
| Normal weight | 1 (reference) |  |
| Overweight | 1.14 (1.06, 1.23) |  |
| Obesity | 1.21 (1.10, 1.33) |  |
| Cohort |  |  |
| 1973-78 | 1 (reference) |  |
| 1989-95 | 1.11 (1.03, 1.19) |  |
| BMI by cohort |  |  |
| Underweight (73-78) |  | 1.60 (1.38, 1.86) |
| Normal weight (73-78) |  | 1 (reference) |
| Overweight (73-78) |  | 1.04 (0.93, 1.17) |
| Obesity (73-78) |  | 0.96 (0.82, 1.13) |
| Underweight (89-95) |  | 1.07 (0.86, 1.32) |
| Normal weight (89-95) |  | 1.13 (1.03, 1.24) |
| Overweight (89-95) |  | 1.37 (1.22, 1.52) |
| Obesity (89-95) |  | 1.48 (1.31, 1.68) |
| Wave |  |  |
| 1 | 1 (reference) | 1 (reference) |
| 2 | 0.84 (0.79, 0.89) | 0.86 (0.81, 0.91) |
| 3 | 0.79 (0.74, 0.84) | 0.83 (0.78, 0.88) |
| Smoker |  |  |
| Never smoked |  | 1 (reference) |
| Ex-smoker |  | 1.33 (1.22, 1.46) |
| Current smoker |  | 1.49 (1.37, 1.62) |
| Ever pregnant |  |  |
| No |  | 1 (reference) |
| Yes |  | 0.86 (0.79, 0.93) |
| Physical activity |  |  |
| Nil/sedentary |  | 1.05 (0.92, 1.20) |
| Low |  | 0.97 (0.88, 1.05) |
| Moderate |  | 1 (reference) |
| High |  | 0.89 (0.83, 0.97) |

Model 1: Additive effects for BMI category and cohort, with adjustment for wave.

Model 4: Interaction effects for BMI category and cohort, with multiple imputation for missing values and adjustment for wave, smoking status, ever having been pregnant and level of physical activity.

Table S11. Odds ratios for ever having a diagnosis of endometriosis

|  | Model 1 | Model 4 |
| --- | --- | --- |
| BMI |  |  |
| Underweight | 0.98 (0.80, 1.20) |  |
| Normal weight | 1 (reference) |  |
| Overweight | 1.07 (0.96, 1.18) |  |
| Obesity | 1.18 (1.04, 1.35) |  |
| Cohort |  |  |
| 1973-78 | 1 (reference) |  |
| 1989-95 | 1.45 (1.25, 1.67) |  |
| BMI by cohort |  |  |
| Underweight (73-78) |  | 0.97 (0.70, 1.33) |
| Normal weight (73-78) |  | 1 (reference) |
| Overweight (73-78) |  | 1.08 (0.92, 1.27) |
| Obesity (73-78) |  | 1.15 (0.92, 1.44) |
| Underweight (89-95) |  | 1.53 (1.13, 2.07) |
| Normal weight (89-95) |  | 1.52 (1.29, 1.80) |
| Overweight (89-95) |  | 1.59 (1.33, 1.90) |
| Obesity (89-95) |  | 1.75 (1.44, 2.12) |
| Wave |  |  |
| 1 | 1 (reference) | 1 (reference) |
| 2 | 2.30 (2.11, 2.51) | 2.27 (2.08, 2.48) |
| 3 | 3.15 (2.87, 3.47) | 3.08 (2.79, 3.39) |
| Smoker |  |  |
| Never smoked |  | 1 (reference) |
| Ex-smoker |  | 1.07 (0.93, 1.22) |
| Current smoker |  | 1.22 (1.07, 1.39) |
| Ever pregnant |  |  |
| No |  | 1 (reference) |
| Yes |  | 1.16 (1.04, 1.29) |
| Physical activity |  |  |
| Nil/sedentary |  | 1.12 (0.97, 1.30) |
| Low |  | 0.99 (0.90, 1.09) |
| Moderate |  | 1 (reference) |
| High |  | 0.99 (0.91, 1.08) |

Model 1: Additive effects for BMI category and cohort, with adjustment for wave.

Model 4: Interaction effects for BMI category and cohort, with multiple imputation for missing values and adjustment for wave, smoking status, ever having been pregnant and level of physical activity.

Table S12. Odds ratios for miscarriage as a proportion of pregnancies, for women who had ever been pregnant.

|  | Model 1 | Model 4 |
| --- | --- | --- |
| BMI |  |  |
| Underweight | 1.11 (0.93, 1.34) |  |
| Normal weight | 1 (reference) |  |
| Overweight | 0.99 (0.88, 1.11) |  |
| Obesity | 1.12 (0.97, 1.28) |  |
| Cohort |  |  |
| 1973-78 | 1 (reference) |  |
| 1989-95 | 0.56 (0.49, 0.65) |  |
| BMI by cohort |  |  |
| Underweight (73-78) |  | 0.96 (0.77, 1.21) |
| Normal weight (73-78) |  | 1 (reference) |
| Overweight (73-78) |  | 1.04 (0.92, 1.17) |
| Obesity (73-78) |  | 1.18 (1.00, 1.39) |
| Underweight (89-95) |  | 0.94 (0.67, 1.33) |
| Normal weight (89-95) |  | 0.61 (0.53, 0.71) |
| Overweight (89-95) |  | 0.57 (0.49, 0.67) |
| Obesity (89-95) |  | 0.65 (0.55, 0.76) |
| Wave |  |  |
| 1 | 1 (reference) | 1 (reference) |
| 2 | 1.29 (1.16, 1.43) | 1.28 (1.17, 1.41) |
| 3 | 1.37 (1.24, 0.82) | 1.37 (1.25, 1.50) |
| Smoker |  |  |
| Never smoked |  | 1 (reference) |
| Ex-smoker |  | 1.19 (1.07, 1.33) |
| Current smoker |  | 1.28 (1.14, 1.43) |
| Physical activity |  |  |
| Nil/sedentary |  | 1.08 (0.95, 1.22) |
| Low |  | 1.02 (0.93, 1.13) |
| Moderate |  | 1 (reference) |
| High |  | 1.12 (1.02, 1.24) |

Model 1: Additive effects for BMI category and cohort, with adjustment for wave.

Model 4: Interaction effects for BMI category and cohort, with multiple imputation for missing values and adjustment for wave, smoking status and level of physical activity. All women included in this analysis had been pregnant at least once.
